# Molecular, Translational and Clinical Research on the Two Most Common Forms of Neurodegenerative Dementia: Alzheimer’s Disease and Dementia with Lewy Bodies

**DOI:** 10.3390/ijms24097996

**Published:** 2023-04-28

**Authors:** Eleonora Napoli

**Affiliations:** Department of Neurology, University of California Davis School of Medicine, Sacramento, CA 95817, USA; enapoli@ucdavis.edu

While not a specific disease, dementia is a term used to describe the deterioration of cognitive function beyond what would be expected because of natural biological aging. It manifests as the worsening or loss of multiple higher cortical functions, including memory, attention, executive function, learning capacity, judgement, language, motor skills, and social cognition, affecting an individual’s ability to perform everyday activities independently [1].

The World Health Organization estimates that the number of individuals with dementia worldwide is approximately 55 million [2], with nearly 10 million new cases every year (one every 3.2 s) and an expectancy of approximately 153 million by 2050 [3]. The global financial burden of dementia was estimated to be USD 1.3 trillion in 2019 and may increase to USD 2.8 trillion by 2030. Alarmingly, the prevalence of early-onset dementia (onset of symptoms before 65 years of age, also known as young-onset dementia, YOD) is also on the rise, with the number of cases doubling every 5 years and an incidence of 42–54 per 100,000 in people aged 30–64 years and 78–98 per 100,000 for those aged 45–64 years [4].

Recently, the cases of actors Robin Williams, who suffered from Lewy body dementia [5] (believed to be a contributing factor to his suicide in 2014), and more recently Bruce Willis, diagnosed with an early-onset form of a neurodegenerative dementia [6,7], have sparked interest and awakened awareness on the debilitating nature of YOD and its consequences on psychological wellbeing, partnerships, parenthood, social life, and occupational functioning [8] not to mention the considerable caregiver burden and delayed access to appropriate care, sometimes leading to long-term misdiagnosis [9,10].

This brief prologue underscores not only the urgency of public health planning and policy to address the needs of this ever-growing population but also the critical role of the scientific community’s efforts aimed at identifying potential new avenues to early diagnose, treat, and even prevent dementia.

A quick search in the NIH-based ClinicalTrials.gov database [https://clinicaltrials.gov/ct2/home (accessed on 22 March 2023)] using the keyword “dementia” resulted in 1051 active (recruiting and non-recruiting) observational (301) or interventional (750) dementia (or dementia-related) clinical trials, involving tens of thousands of institutions and centers worldwide.

As clichéd a statement as this is, research efforts aimed at defining the pathogenetic mechanisms underlying neurodegenerative disorders leading, directly or indirectly, to dementia are an essential requirement for identifying new pharmacological targets and neuroprotective treatments.

Of the 15 papers published in this Special Issue of the *International Journal of Molecular Sciences*, a dozen report current molecular, translational and clinical research findings on Alzheimer’s disease (AD) [11,12,13,14,15,16], dementia with Lewy bodies (DLB) [17] and other neurological disorders such as FXTAS [18] and stroke [19,20,21,22] that share somewhat overlapping cognitive deficits with AD and DLB [23,24,25,26,27,28,29].

Although all these reports are noteworthy and provide novel insights and original outlooks on the most recent advances in the field of neurological disorders, for brevity’s sake, the following part of this editorial will focus on the ones discussing the two most common neurodegenerative causes of dementia: AD and DLB [30].

From a pathomechanistic standpoint, AD is characterized by extracellular amyloid-beta (Aβ) plaques and intracellular neurofibrillary or extraneuronal ghost tau tangles in the brain, affecting primarily the medial temporal cortex and the neocortical association areas and accounting for the predominant disturbances of episodic memory. Conversely, the neuropathology of DLB centers on neuronal loss and the presence of Lewy bodies (constituted mainly by α-synuclein) in the subcortical nuclei and the frontal and parietal lobes, accounting for alertness, awareness, executive, and visuospatial deficits [31]. Since its recognition as a neurodegenerative disorder, a body of research has focused on the differentiation of DLB from AD. DLB is associated with a higher mortality rate, greater behavioral disturbances, lower quality of life, and higher resource needs compared with AD [32,33,34].

The research group led by Dr. Santamaria [17] characterized the specific molecular mechanisms underlying the process of neurodegeneration at the level of primary olfactory areas in postmortem samples from elderly subjects with DLB. As acute olfactory abnormalities are associated with DLB progression and α-synuclein propagation from the olfactory bulb (OB) has been proposed as a potential onset of this pathology, the authors have carried out an in-depth quantitative proteomic study in combination with functional interaction data and biochemical approaches in order to determine the dysregulation of the OB proteome in DLB. Overall, the protein interactome mainly comprised specific protein clusters related to translation, NMDA receptor effectors, synaptic vesicle cycle, and an innate immune system. The observed OB protein homeostasis imbalance would ultimately affect translation-related processes, as shown by the significant downregulation of several ribosomal proteins. The findings in this study shed some light on the role played by olfactory structures in the pathophysiology of DLB and identified protein mediators that may potentially be used as biomarkers for early DLB diagnosis and evolution or as potential therapeutic targets.

A rapidly growing literature strongly suggests that exercise, more specifically aerobic exercise, may reduce cognitive impairment and the risk of developing dementia. Starting from this premise, Yu and colleagues [16] document the effects of 12-week aerobic treadmill exercise on learning and memory capacity, adult hippocampal neurogenesis (AHN), and amyloid precursor protein (APP) proteolytic-pathway-related factors in the APP/PS1 mouse model of AD. The authors showed that treadmill exercise promoted APP cleavage via a non-amyloidogenic pathway, improved the hippocampal microenvironment (by favoring neurogenesis, impairing toxic Aβ deposition, and boosting neuronal survival and differentiation), and enhanced AHN in AD mice, thus improving spatial learning and memory. These findings support the idea that physical exercise-driven neurogenesis may offer hippocampal protection and become a potential disease-modifying treatment for AD and brain aging.

The paper by Jantrapirom et al. [12] highlights the impact of insulin resistance in the exacerbation of AD via its role in regulating amyloid β-protein peptides and the generation of NFTs. By using SH-SY5Y cells, an established and widely used human neuroblastoma cellular model of AD, they found that a 24 h treatment with liraglutide, a glucagon-like peptide-1 (GLP-1) known to be effective at improving peripheral insulin resistance, alleviated neuronal insulin resistance and reduced beta-amyloid formation and tau hyperphosphorylation. This study also showed that neuronal insulin-resistant conditions correlated with an increased expression of pro-apoptotic protein Bax and a decreased expression of anti-apoptotic protein Bcl-2. However, cell exposure to liraglutide did not rescue Bax and Bcl-2 dysregulation in the neuronal insulin-resistant condition, at least at the time point used for their experiments (24 h).

Ex vivo observations of senile plaques that are highly enriched in zinc have recently introduced the possibility that complex crosstalk between metal ions and AD pathological proteins might exist, involving a close relationship between protein misfolding, aggregation, and metal ion homeostasis, particularly zinc. Several recent studies focus on the effect of zinc on Tau physiology and aggregation. One such study, by Moreira and colleagues [13], investigated the interaction of full-length human Tau with calcium and zinc, combining multiple biophysical and mass spectrometry approaches and unveiling new clues in the implication of these two ions in AD’s pathomechanism. The authors established that Tau binds four Zn^2+^ and one Ca^2+^ per monomer, without leading to substantial conformational changes in the intrinsically disordered Tau. However, Tau aggregation is found to proceed differently in calcium- and zinc-bound forms. While the rate of aggregation greatly increased in both instances, the reaction proceeds via fundamentally different mechanisms based on the binding site’s affinity within the Tau sequence that prompts both the rapid formation of seeding oligomers via interactions at high-affinity sites within the repeat domains, as well as amorphous aggregation via low-affinity interactions with residues located elsewhere within the sequence.

Finally, two review articles discuss the potential roles of melatonin (Roy et al. [14]) and stem cell therapy (Chan et al. [11]) in the prevention and treatment of AD.

As described by Roy and colleagues [14], the decreased secretion of the hormone melatonin from the pineal gland has been deemed responsible for circadian dysregulation, sleep disturbances, and declined cognitive function observed in AD patients. Consequently, testing whether melatonin could be an effective resynchronizing agent (or chronobiotic) to improve an AD patient’s condition does not seem farfetched. Some reports have postulated a critical neuroprotective role for melatonin due to the inhibition of Aβ plaques synthesis. Others claim that it mitigates the cholinergic system disturbances observed in AD via the inhibition of AChE release, thereby effectively acting as an acetylcholine enhancer. However, a very limited number of studies have investigated the effects of melatonin on major neurotransmitters or carried out comparisons with other cholinergic drugs.

Although the literature published to date suggests that melatonin could be a potential treatment for AD, the mechanisms that account for its therapeutic effects are still elusive and not widely pursued. Indeed, a search on ClinTrials.gov using the keywords “Alzheimer’s disease” (or “dementia”) and “melatonin” returned a total of six currently recruiting studies.

The review by Chan and colleagues [11] offered an overview of the mechanisms, therapeutic potential, current preclinical and clinical research status, and limitations of stem cell therapy.

While pharmacological therapy that could revert AD continues to be the holy grail of medical researchers, alternative approaches are also being pursued, including the use of stem cell therapy. Stem cell treatment has been employed successfully in some AD animal models. Recent preclinical studies have produced promising preliminary data, and some human clinical trials are ongoing.

It is outside the scope of this editorial to delve into the biology and potential therapeutic mechanisms of stem cells, even in the context of AD or other types of dementia; a large body of scientific literature is available on the subject. However, one aspect that is often disregarded when discussing stem cell therapy (or at least outweighed by the excitement brought about by the potential promises of this field of research) is the notion that, as the authors briefly mentioned, should stem cells be proven to be useful in the fight against AD, this therapy will likely not be readily available to much of the population due to its prohibitive costs. It is estimated that in 2023 stem cell therapy costs range anywhere from USD 5k–50k. Treatment cost varies dramatically based on a variety of factors including, but not limited to, the condition being treated; source; type; quality and the number of stem cells administered; expenses related to the laboratory work for cell expansion; and all other indirectly associated costs (i.e., costs of delivery—which may need to include neurosurgical intracranial implantation—inpatient/hospitalization charges, etc). Another critical aspect to consider is the issue raised by some studies that stem cell proliferation might not progress when exposed to an aging “system” [35], and although stem cells remain active into old age, changes in the surrounding microenvironments may inhibit their regenerative potential [36]. This would pose a significant obstacle to the application of stem cell therapy in AD and other advanced neurodegenerative disorders.

Both reviews emphasize that no disease-modifying pharmacotherapies for AD exist to date and that the available medications (namely cholinesterase inhibitors and memantine) can only temporarily slow the progression of the disease, partly relieving some dementia symptoms, with a limited improvement in the quality of life and mostly in early to moderate cases.

At the time these papers were accepted for publication in this *IJMS* Special Issue, this statement was spot on, and in some measure, it still is. However, since then, two AD treatments have been approved by the FDA under the agency’s accelerated approval pathway, both of which target the pathophysiological processes underlying AD. The first one, aducanumab, a human monoclonal antibody with high specificity to Aβ soluble oligomers and insoluble fibers, was approved in the summer of 2021 [37]. In January of 2023, another humanized monoclonal antibody that binds with high affinity to soluble Aβ protofibrils, lecanemab [38], was granted FDA-accelerated approval. In three phase 3 randomized, double-blind, and placebo-controlled clinical trials (EMERGE and ENGAGE (parallel group studies for aducanumab), and Clarity AD (lecanemab)), both drugs produced a marked dose-dependent reduction in brain amyloids and a slower decline on clinical outcome measures upon the administration of the approved doses (6–10 mg/kg and 10 mg/kg, respectively) over a time period of, respectively, 78 and 72 weeks [38] in 1600 and 1795 amyloid-positive participants with early-stage AD. However, the limited—although statistically significant—efficacy on cognitive decline (slowed by a mere 27% for both drugs), numerous adverse effects (mild to moderate infusion-related reactions, amyloid-related image abnormalities with cerebral microhemorrhages, cerebral macrohemorrhages, or superficial siderosis and headache) and astronomical costs (> USD 28K for a year of treatment [39]) hampered enthusiasm about these drugs. Furthermore, additional efficacy and safety data in post-marketing studies of aducanumab has led to considerable controversy [40], thereby also casting a long shadow over the use of lecanemab.

Currently, a fourth phase 3 clinical trial (TRAILBLAZER-ALZ 2) is ongoing for donanemab, another humanized IgG1 monoclonal antibody that recognizes a pyroglutamate form of Aβ that is aggregated in amyloid plaques. Differently from the former two molecules, which bind various soluble or insoluble species but have low affinity to deposited amyloid plaques, donanemab targets deposited plaque itself, with the potential of clearing existing amyloid burden from the brain, rather than merely preventing the deposition of new plaques or the growth of existing ones [41]. Request for an accelerated approval of donanemab was denied by the FDA in January of 2023 due to the limited number of subjects involved in the trial, but applications for traditional FDA approval remain on track for mid-2023.

This editorial aimed to summarize the findings collected in this Special Issue, providing some context for neurodegenerative dementia disorders and related therapeutic approaches.
